# Terminal-Repeat Retrotransposons in Miniature (TRIMs) in bivalves

**DOI:** 10.1038/s41598-019-56502-y

**Published:** 2019-12-27

**Authors:** Eva Šatović, Andrea Luchetti, Juan J. Pasantes, Daniel García-Souto, Andrea Cedilak, Barbara Mantovani, Miroslav Plohl

**Affiliations:** 1 Division of Molecular Biology, Ruđer Bošković Institute, Zagreb, Croatia; 2 Dipartimento di Scienze Biologiche, Geologiche e Ambientali, Università di Bologna, Bologna, Italy; 3 Departamento de Bioquímica, Xenética e Inmunoloxía, Universidade de Vigo, Vigo, Spain; 4 Department of Zoology, Genetics and Physical Anthropology, Universidade de Santiago de Compostela, Santiago de Compostela, Spain; 5 Cancer, Ageing and Somatic Mutation, Wellcome Sanger Institute, Hinxton, Cambridgeshire CB10 1SA UK

**Keywords:** Molecular evolution, Evolutionary biology, Transposition

## Abstract

Terminal repeat retrotransposons in miniature (TRIMs) are small non-autonomous LTR retrotransposons consisting of two terminal direct repeats surrounding a short internal domain. The detection and characterization of these elements has been mainly limited to plants. Here we present the first finding of a TRIM element in bivalves, and among the first known in the kingdom Animalia. Class Bivalvia has high ecological and commercial importance in marine ecosystems and aquaculture, and, in recent years, an increasing number of genomic studies has addressed to these organisms. We have identified biv-TRIM in several bivalve species: *Donax trunculus*, *Ruditapes decussatus, R. philippinarum*, *Venerupis corrugata, Polititapes rhomboides, Venus verrucosa, Dosinia exoleta, Glycymeris glycymeris, Cerastoderma edule, Magallana gigas, Mytilus galloprovincialis*. biv-TRIM has several characteristics typical for this group of elements, exhibiting different variations. In addition to canonically structured elements, solo-TDRs and tandem repeats were detected. The presence of this element in the genome of each species is <1%. The phylogenetic analysis showed a complex clustering pattern of biv-TRIM elements, and indicates the involvement of horizontal transfer in the spreading of this element.

## Introduction

Eukaryotic genomes display many transposable elements (TEs) that can be grouped into two main classes according to their mechanisms of transposition. Class I elements transpose by RNA-mediated “copy-and-paste” mechanisms, whilst Class II elements spread through DNA-mediated processes which can be classified as “cut-and-paste”, “peel-and-paste”, and “self-synthesizing”^1–4^. Each class holds autonomous and non-autonomous copies; the first ones are able to produce all enzymes required for their transposition whereas the latter depend on those produced by autonomous partner elements. Based on their structure, Class I elements are further divided into two major subclasses, Long Terminal Repeat (LTR) and non-LTR retrotransposons^5^. Terminal repeat retrotransposons in miniature (TRIMs) are small (less than 1000 bp) non-autonomous LTR retrotransposons. They contain terminal direct repeats (TDRs) of 100–250 bp, equivalent to LTRs, flanking an internal domain which starts with a primer binding site (PBS), complementary to a tRNA, and ends with a polypurine tract (PPT)^6^. These elements were originally described in potato and *Arabidopsis*^6^ and later found in many more plant genomes, from land plants to algae^7–10^. TRIM elements seem to be involved in active restructuring of plant genomes by affecting their promoters and coding regions. They can be also implicated in transduction of host genes or serve as hotspots for further retrotransposon insertions^6^. Their potential to be used as molecular markers has been exploited in plants^11–14^ and fungi^15^. In contrast, the only reports to date of TRIM elements within the animal kingdom are in the red harvester ant *Pogonomyrmex barbatus*^16^, in Taeniid cestodes^17^ and in the honey bee *Apis mellifera*^18^.

Bivalves are a large class of marine and freshwater mollusks of high importance in marine ecosystems and aquaculture. In accordance with their significant ecological and commercial value, the interest in genome research on these organisms is growing steadily, with an increasing number of genomic projects being performed (19 available in NCBI GenBank database, September 2019). So far, only a few non-autonomous mobile elements in bivalve mollusks have been described in detail; one of them belonging to Class I^19^ and a few belonging to Class II^20–23^.

Here, we report on the discovery and characterization of a new TRIM element, biv-TRIM, identified in 11 bivalve species belonging to two different Bivalvia subclasses, Heterodonta and Pteriomorphia. This is the first report of a TRIM in bivalve mollusks.

## Results

### biv-TRIM detection and general features

**Figure 1:**
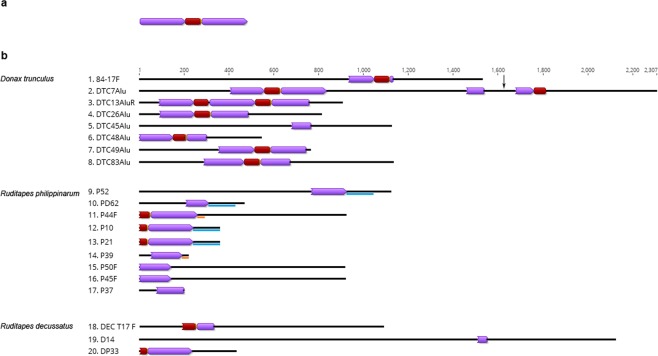
Schematic presentation of the canonical biv-TRIM element (**a**) and the genomic fragments from *D. trunculus, R. philippinarum and R. decussatus* obtained by colony lift (**b**). TDRs are labeled in purple and inner domains in dark red. Similarities in sequence segments following TRIM elements in clones P52, P10, P21, PD62 are labelled blue while in P44F and P39 are labelled orange. Location of the insertion of an unrelated sequence into TRIM TDR domain in clone DTC7Alu has been labeled with a black arrow.

Small local databases of genomic fragments enriched in repetitive DNAs of *Donax trunculus*, *Ruditapes philippinarum* and *R. decussatus* were generated previously, as described in Materials and Methods section. Initial “all-*versus*-all” blast search carried out within 194 genomic fragments of these species (96 belonging to *Donax trunculus*, 36 to *Ruditapes philippinarum* and 62 to *R. decussatus*) resulted in sequence groupings based on high-scoring segment pairs, suggesting the presence of highly similar nucleotide sequences dispersed throughout different genomic environments. One of the obtained groups of sequences consisted of 20 genomic fragments (eight of *D. trunculus*, nine of *R. philippinarum* and three of *R. decussatus*, presented in Fig. 1). The examination of their sequences revealed the presence of several structural features (TDRs, inner domains, PPTs) characteristic of TRIM elements, as described by Witte *et al*.^6^ and presented in Fig. 1a. *D. trunculus* clones holding complete elements were employed for element type determination, while clones showing truncated copies were attributed to their corresponding element based on sequence similarity. Database search could not reveal similarity of this element with any other known TRIM. Therefore, mobile elements depicted in this work were named biv-TRIM. In the studied sample, genomic fragments of *D. trunculus* were found to harbor copies with canonical structure together with truncated and rearranged elements. Moreover, a biv-TRIM observed in the DTC13AluR clone demonstrated that this element can be also repeated in tandem (Fig. 1b).

A primer pair was designed from the DNA sequence of biv-TRIM elements derived from *D. trunculus*. PCR amplification yielded additional copies of the whole element and/or single TDR domains from *D. trunculus*, *R. philippinarum* and *R. decussatus* as well as from eight additional bivalve species (*Venerupis corrugata, Polititapes rhomboides, Venus verrucosa, Dosinia exoleta, Glycymeris glycymeris, Cerastoderma edule, Magallana gigas, Mytilus galloprovincialis*), while in *Ostrea edulis, Spisula solidissima, Mytilopsis sallei, Congeria kuscheri* and *Dreissena polymorpha* the element was not detected. The complete list and taxonomic status of the species used in this work is shown in Table 1. The description of all identified biv-TRIM sequences is shown in Supplementary Table S3. The examined biv-TRIM elements displayed 202–218 bp TDRs, and a 77–80 bp internal domain with a terminal 7 bp PPT. The PPT sequence was GGGGAGA for most of the isolated biv-TRIMs.

**Table 1:** Bivalve species tested for the presence of biv-TRIM element. Species in which TRIM element was not detected are underlined.

Subclass	Order	Family	Genus	Species	Former name
Heterodonta	Cardiida	Donacidae	*Donax*	*Donax trunculus* (Linnaeus, 1758)	
		Cardiidae	*Cerastoderma*	*Cerastoderma edule* (Linnaeus, 1758)	
	Venerida	Veneridae	*Ruditapes*	*Ruditapes decussatus* (Linnaeus, 1758)	
				*Ruditapes philippinarum* (Adams & Reeve, 1850)	
			*Venerupis*	*Venerupis corrugata* (Gmelin, 1791)	*Venerupis pullastra*
			*Polititapes*	*Polititapes rhomboides* (Pennant, 1777)	*Venerupis rhomboides*
			*Venus*	*Venus verrucosa* (Linnaeus, 1758)	
			*Dosinia*	*Dosinia exoleta* (Linnaeus, 1758)	
	Myida	Dreissenidae	*Dreissena*	*Dreissena polymorpha* (Pallas, 1771)	
			*Mytilopsis*	*Mytilopsis sallei* (Récluz, 1849)	
			*Congeria*	*Congeria kusceri* (Bole, 1962)	
		Mactridae	*Spisula*	*Spisula solidissima* (Dillwyn, 1817)	
Pteriomorphia	Ostreida	Ostreidae	*Magallana*	*Magallana gigas* (Thunberg, 1793)	*Crassostrea gigas*
			*Ostrea*	*Ostrea edulis* (Linnaeus, 1758)	
	Arcida	Glycymerididae	*Glycymeris*	*Glycymeris glycymeris* (Linnaeus, 1758)	
	Mytilida	Mytilidae	*Mytilus*	*Mytilus galloprovincialis* (Lamarck, 1819)	

Search for PBS sites was done by blasting inner domains of biv-TRIMs against eukaryotic tRNA sequences from publicly available database and tRNAs from 3 bivalve species (*R. philippinarum, Magallana gigas, Mytilus galloprovincialis*). This search resulted in short segments of similarity, but varying in their position within the inner domain of the TRIM element, and within the tRNA. For *Glycymeris glycymeris* and *Venus verrucosa* elements similarity to tRNA^Tyr^ has been detected immediately downstream of the 5′ TDR, but corresponding to the central part of the tRNA. Therefore, despite the similarities detected within the TRIM inner domain, canonical PBS site, could not be unambiguously identified (Fig. S1). However, inner domains harbour inverted repeats, palindromic sequences or conserved segments. For example, in *D. trunculus, C. edule* and *M. gigas* 9-bp, and in *M. galloprovincialis* 10-bp long inverted repeats are 25 bp apart, allowing the formation of a hairpin structure, whose central part shows similarity to tRNA^Gln^ while similarity to tRNA^Leu^ overlaps with the inverted repeat. In addition, inner domains of all species (except *V. corrugata*) have conserved TGATCCCAAAGGAC segment in the central part that is positioned between the two inverted repeats or following palindromic structures (Fig. S1).

Sequence divergence among complete biv-TRIM TDR domains obtained by colony lift and PCR amplification ranged from complete identity up to 50% divergence, with an average inter-specific divergence of 26.7%. In addition, part of the colony lift-obtained genomic fragments (Fig. 1b) allowed the inspection of the sequences surrounding the biv-TRIM elements. In the case of *D. trunculus* and *R. decussatus* there was no similarity among sequences preceding or following the TRIM elements, either within the same or among different fragments. In contrast, some similarities were found in sequences following the *R. philippinarum* elements (116 bp segments of P52, P10, P21, PD62 with average similarity of 87% (P10 and P21 showing 100% similarity in this segment) and 28 bp stretches with 86% similarity between P44F and P39).

### Search for biv-TRIM in sequenced bivalve genomes

**Figure 2:**
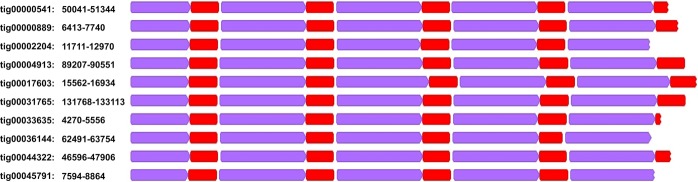
Tandem arrays within the ten randomly chosen contigs of the *R. philippinarum* draft genome. Genomic coordinates refer to the position of the array within the contig. TDRs are labeled in purple and inner domains in red.

Search for biv-TRIM was performed in the draft genome of *R. philippinarum*^24^, as well as in the genomic data available for other 19 bivalve species (*Argopecten irradians, Pinctada imbricata, Saccostrea glomerata, Modiolus philippinarum, Bathymodiolus platifrons, Limnoperna fortunei, Corbicula fluminea, Dreissena polymorpha, Mizuhopecten yessoensis, Mytilus galloprovincialis, Crassostrea gigas, Bankia setacea, Venustaconcha ellipsiformis, Crassostrea virginica, Sinonovacula constricta, Lutraria rhynchaena, Dreissena rostriformis, Venustaconcha ellipsiformis, Argopecten irradians concentricus*) in GenBank. BLAST on *R. philippinarum* draft genome resulted in 46,567 positive hits, with high scoring segment pairs ≥100 bp, distributed over 12,770 contigs (39.4% of the total number of contigs). Of these, only 2,458 contained a single biv-TRIM BLAST hit, while the remaining contigs harboured from 2 to 81 hits, either interspersed or contiguous. To avoid false overlapping hits caused by the presence of TDRs, contiguous multiple BLAST hits were merged into single spans. This resulted in 27,489 biv-TRIM genomic insertion loci, ranging from 100 bp to 16,618 bp, with a median length of 126 bp. This indicates that the majority of biv-TRIM insertion loci (96%) were composed of small fragments, shorter than a single copy. Insertion loci containing arrays of biv-TRIM (Fig. 2) followed the tandemization pattern explained in^8^: TDR_x__I_(x–1)_, where I is the inner domain. Therefore, based on this pattern, the longest insertion locus is in the form TDR_58__I_57_. Overall, 5,140,220 bp were covered by biv-TRIM hits, which constitutes about ~0.26% of the sequenced genome (1.9 Gb^24^).

Among 19 other sequenced bivalve genomes available in GenBank, the search yielded partial similarities with biv-TRIM in *Limnoperna fortunei, Corbicula fluminea, Dreissena rostriformis* and *Magallana gigas*. In addition, Transcriptome Shotgun Assembly (TSA) database searches revealed transcripts spanning the complete elements, or parts of them, in *Ruditapes philippinarum, R. decussatus, Paphia undulata, Eurhomalea rufa, Phacoides pectinatus, Panopea globosa* and *Limecola balthica balthica*. In this regard, *in silico* analysis broadened the potential distribution of biv-TRIM retroelements to additional species within the Bivalvia subclasses Heterodonta and Pteriomorphia. The search for LTR retrotransposons that could be involved in TRIM mobility was performed via GenBank and Repbase database (https://www.girinst.org/censor/index.php), but no potential autonomous partner elements have been detected.

### biv-TRIM phylogenetics

**Figure 3:**
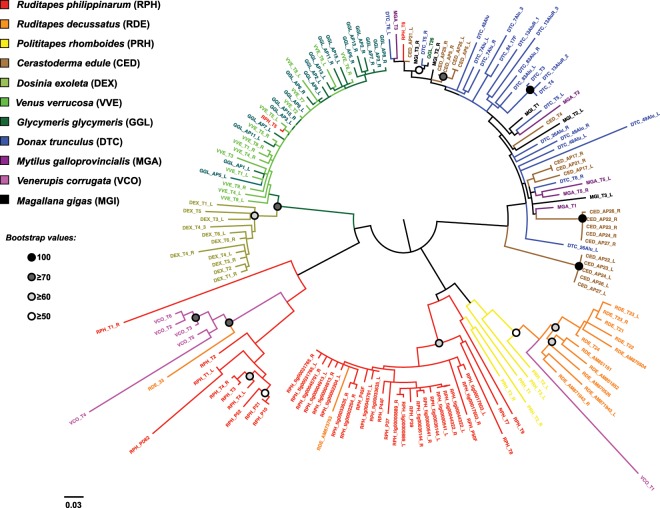
Maximum Likelihood phylogenetic analysis of TRIM elements based on TDR domains. Dots at nodes represent bootstrap values as reported in the legend.

To explore the evolution of biv-TRIM sequences, TDRs and internal domains were used in separate phylogenetic analyses due to the fact that some elements contained single or multiple TDR domains. Maximum Likelihood phylogenetic analysis on 160 TDR domains of the sequenced TRIM elements, including 10 elements randomly selected from the *R. philippinarum* genome, revealed complex clustering patterns (Fig. 3). TDRs from biv-TRIM of *D. exoleta*, *V. verrucosa* and *G. glycymeris* form a single cluster showing two sub-clusters, one harbouring only the *D. exoleta* sequences and the other containing intermingled *V. verrucosa* and *G. glycymeris* ones. The latter sub-cluster also includes one *R. philippinarum* TDR (RPH_T5). TDRs from *R. decussatus* and *V. corrugata* elements were mostly grouped in species-specific clusters. The majority of *R. philippinarum* sequences were included in a supported cluster, while the remaining ones were either scattered along the tree or grouped in an unsupported cluster. TDRs from *C. edule* were grouped in multiple species-specific clusters, scattered among sequences derived from other species. Interestingly, in one of the clusters, left and right TDRs of the two elements CED_AP5 and CED_AP25 were grouped together, whereas left and right TDRs of other five elements (CED_AP22, CED_AP23, CED_AP24, CED_AP26, CED_AP27) were consistently grouped in two different clusters (Fig. 3). The TDRs of the remaining *C. edule* biv-TRIM elements as well as those of the remaining species were found interspersed without any remarkable clustering pattern.

**Figure 4:**
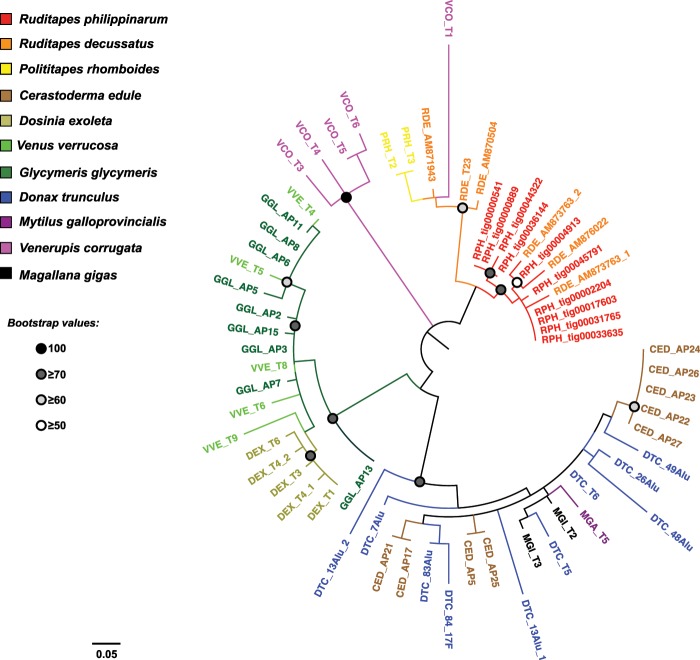
Maximum Likelihood phylogenetic analysis of TRIM elements based on inner domains. Dots at nodes represent bootstrap values as reported in the legend.

The phylogenetic analysis based on 64 inner domains of biv-TRIM elements followed the same distribution pattern (Fig. 4).

### Genomic contributions of biv-TRIM

The abundance of the biv-TRIM elements in the genome of each species was assessed by dot blot hybridization (Fig. S2). The obtained values showed a low representation of biv-TRIM elements in six of the species (0.65% for *D. exoleta*, 0.20% for *R. philippinarum* and *P. rhomboides*, 0.05% for *V. verrucosa* and *D. trunculus* and 0.03% for *R. decussatus*), while amounts in the remaining five (*M. gigas, G. glycymeris, M. galloprovincialis, V. corrugata* and *C. edule*) were significantly lower and could not be determined.

### biv-TRIM cytogenetics

Multiple trials were performed in the attempt to determine chromosomal localization of biv-TRIM elements in the eleven bivalve species. Despite of different stringency conditions employed in the hybridization experiments, there was no reproducibility in the hybridization signals even in species with the highest TRIM contribution (Fig. S3). For control purposes, another repetitive sequence, H3 histone genes, was also mapped to metaphase chromosomes. As the FISH protocols employed in this study were optimized for bivalve chromosomes and gave good results when used to detect repetitive sequences in these organisms^25–27^, including H3 controls in this work (Fig. S3), the lack of clear TRIM signals can be interpreted as a result of low copy-number and predominantly scattered organization pattern of these elements in the examined genomes.

## Discussion

Although frequently found in plants^6,7,9–14^, there are only a few reports on the presence of TRIM elements in the kingdom Animalia^16–18^. This work is the first report of TRIM element in the class Bivalvia. biv-TRIM elements have been detected in 11 species belonging to subclasses Heterodonta and Pteriomorphia, while its presence could not be confirmed in 5 species, belonging to the same subclasses (Table 1). Possible reasons could be either absence of TRIM elements in these genomes or that their sequences are incomplete or too divergent to be detected. If we assume a strictly vertical inheritance of this element, the age of biv-TRIM would correspond to the estimated time of diversification of these bivalve subclasses, dating back to the Cambrian, about 516 Ma^28^. The estimated time of origin is very close to that of the BIV160 satellite DNA (540 Ma^29^), the DTHS3 satellite DNA (516 Ma^30^) and the SINE element RUDI (>550 Ma^19^). Altogether, these data suggest that both tandem and interspersed repetitive sequences might be present in bivalve genomes during long evolutionary periods. Despite the proposed old age of this element, the intraspecies nucleotide sequence similarity of biv-TRIM variants is remarkable within *D. exoleta, V. verrucosa* and *G. glycymeris*, 93.9%, 93.9% and 96.5%, respectively. At the same time, copies derived from *V. verrucosa* and *G. glycymeris* show also high interspecies similarity, remaining unresolved and forming a single, monophyletic group in the phylogenetic analysis (Fig. 3), although these species belong to different subclasses (Heterodonta and Pteriomorphia, Table 1). Most parsimonious explanation would be *de novo* acquisition of the element through horizontal transfer (HT), a mechanism already known to be included in the propagation of mobile elements among many organisms^19^. HT in bivalves and other aquatic species of multiple phyla was proposed to be particularly facilitated, due to the aquatic environment^31^. As both *D. exoleta* and *V. verrucosa* belong to the family Veneridae (Table 1), grouping of biv-TRIM copies from these two species is not surprising. However, since *V. verrucosa* elements were clustered with those from *G. glycymeris* (Fig. 3), and the two species show very high nucleotide similarity among their biv-TRIM elements (Fig. S4), the most probable explanation is HT going from *V. verrucosa* to *G. glycymeris*. In this regard, it is worth mentioning that these two species show unexpected similarity not only in biv-TRIM retroelements but also in tandemly repeated sequences, specifically, BIV160 satellite DNA. BIV160 monomers isolated from the genomes of these two species exhibit significant sequence similarity (97.7%), and group together in the phylogenetic analysis^29^. Since BIV160 monomers show sequence similarity to internal repeats of non-autonomous *pearl* element^29^ belonging to the Helentron superfamily of rolling circle transposons^32^, it is not excluded that both types of mobile elements were transferred horizontally in these two species.

Elements from the rest of the analyzed species showed a mixture of clustered species-specific copies and scattered sequences, or are just scattered across the tree without any apparent grouping. This could be possibly linked to recombination processes, accumulation of mutations and/or HT events.

While a typical TRIM retrotransposon contains two TDRs flanking an internal domain, some TRIM elements can be more complex, containing increased numbers of TDRs and internal domains repeated in tandem. Such tandemly arranged TRIMs are frequently found in plant genomes^8,10^. In the animal kingdom, tandemly repeated TRIMs together with solo-TDRs have been reported in taeniid cestodes^17^. For PbTRIM in ants no tandem organization was reported, although a large number of solo-TDRs exist^16^. In genomic fragments of *D. trunculus* and *R. philippinarum* we have observed the presence of both solo-TDRs and tandemly arranged biv-TRIMs, together with canonically structured elements. Propensity of transposable elements to form tandem arrays has been noticed in bivalve (and other) genomes, reviewed in^33^. Yin *et al*.^9^ proposed a mechanism involved in generating elements with several LTRs. According to this mechanism, closely positioned elements experience a DNA recombination event between 5′ LTR of one element and 3′ LTR of the other. The difference among the TDRs, which are now organized in tandem, has been introduced, due to the inter-element unequal recombination. For example, the diversity of left and right TDRs in PCR-obtained *C. edule* biv-TRIM could be explained by recombination events between different elements. Tandem arrangement of elements and generation of solo-TDRs as a consequence of a high rate of recombination events between intact elements has also been suggested by Liu *et al*.^10^. Also, Vitte and Panaud^34^ proposed that solo-LTRs, which are frequent derivatives of LTR-retrotransposons, originate from unequal crossing-over events between two LTRs of a single element. In the case of the solo-TDR domain in the DTC7Alu genomic fragment of *D. trunculus*, as well as in several other biv-TRIM sequences, insertion of an unrelated sequence can be noticed (Fig. 1, Table S3). Multiple insertions are a common feature of many repetitive sequences, and TRIM elements are known to serve as hotspots for further insertions^6^. For example, 11% of the Wukong TRIM elements from maize hold insertions of other transposable elements, including hAT family DNA transposon and *Copia*-like LTR retrotransposon^10^.

Search for autonomous elements showing sequence similarity with biv-TRIM element did not reveal autonomous partner elements in publicly available databases, but it must be mentioned that sequenced genomes are available only for two (*Magallana gigas* and *Mytilus galloprovincialis*) out of 11 bivalve species in which biv-TRIM has been detected (Table 1). In other studied organisms, putative autonomous partner elements have been proposed for some TRIM elements^8,10,17^, while the inability to detect autonomous variants has been reported for several others^6,12,16^.

Typical PBS sites, usually positioned immediately downstream of the 5′ TDR, could not be identified in biv-TRIM elements. However, different substructures and conserved segments within the inner domain were detected (Fig. S1). An absence of PBS has been observed also in fungal GalEa *Copia* retrotransposons^35^. There, 9-bp inverted repeats also can be found in inner domains, distant to each other from 12 bp to 41 bp, similarly as in biv-TRIM from several species. Another similarity is that inverted repeats CHSeq1 and CHSeq2 found in inner domains of GalEa elements regularly show a mismatch between the nucleotide 7 from CHSeq1 and the nucleotide 3 from CHSeq2, as also observed for the inverted repeats found in the biv-TRIM inner domains. Such segments were proposed to form a hairpin structure in GalEa elements (Conserved Hairpin Site) and the authors hypothesize that it could act as a PBS in the transposition cycle, although no clear connection with any of previously described models has been established yet^35^. Lack of conventional PBS has also been noticed in Tf1, Tf2, Cft-1, Maggy, Skippy, Boty, Grasshopper and Afut1 LTR retrotransposons, which use a self-priming mechanism to initiate synthesis of reverse transcripts, instead of a PBS derived from tRNA^36^. For biv-TRIM element no clear mechanism can be proposed, although there is a possibility that different substructures, conserved segments and sequence similarity to tRNAs within the inner domain could be included in retrotrasposition process unrelated to the conventional PBS site.

The genomes of the 11 bivalve species examined in this work showed notable differences in the abundance of TRIM retrotransposons. A general feature, noticeable in all analyzed species, is that the presence of this element in the genome is low, <1%. A similar situation can be found in *Apis mellifera* where TRIM elements constitute 0.99% of the genome^18^. In plants, Cassandra TRIM elements make up approximately 0.8% of the pear, 0.63% of the apple, 0.17% of the peach, 0.1% of the mei, and 0.03% of the woodland strawberry genome^9^. Very low genome contribution of the TRIM element in the examined bivalve species, together with predominantly interspersed organizational pattern and existence of a number of divergent variants, resulted in the inability to localize these elements on the chromosomes.

This work brings the first finding of a TRIM element in bivalve molluscs, which is the fourth case in the animal world reported so far. biv-TRIM exhibits most of the structural characteristics typical for this group of retroelements, in addition to which solo-TDRs and tandemly repeated forms can be found. The presence of this element in species belonging to the subclasses Heterodonta and Pteriomorphia would suggest that biv-TRIM is an ancient element, dating back to the Cambrian in a strictly vertical inheritance scenario. However, phylogenetic analysis, showing complex clustering pattern of biv-TRIM elements, indicates possibility of its spread by multiple, and probably frequent bursts of HT events, and would imply a more recent origin of this element in some species.

## Materials and Methods

### Biological samples and DNA isolation

Adult specimens belonging to the species: *Donax trunculus*, *Ruditapes philippinarum*, *R. decussatus*, *Glycymeris glycymeris*, *Dosinia exoleta*, *Venus verrucosa*, *Venerupis corrugata*, *Polititapes rhomboides*, *Mytilus galloprovincialis*, *Cerastoderma edule*, *Magallana gigas* and *Spisula solidissima* originate from Galicia (Spain), *Ostrea edulis* from the Adriatic (Croatia), *Congeria kuscheri* from the Dinaric Karst (Croatia), *Dreissena polymorpha* from lake Jarun (Croatia), and *Mytilopsis sallei* from Hong Kong (China). Genomic DNA was isolated from adductor muscles using both standard phenol-chloroform protocol and DNeasy Blood & Tissue Kit (QIAGEN).

### Partial genomic libraries and colony lift

During a comprehensive study on repetitive DNAs in bivalves, small libraries of genomic fragments enriched in repetitive DNAs were obtained for *D. trunculus*, *R. decussatus* and *R. philippinarum*, as described in Šatović and Plohl^22,37^. To generate partial genomic libraries of three species, 10 μg of genomic DNA of each species was partially digested with 5 U of AluI restriction endonuclease for 5 min at 37 °C. The obtained fragments were ligated into the pUC19/SmaI vector. Following transformation, *E. coli* DH5α competent cells (Invitrogen) were grown on ampicillin-selective plates. Colonies were transferred on 90 mm positively charged membranes (Amersham) and probed with AluI-digested digoxigenin-labeled genomic DNA of the corresponding species. This approach is based on the fact that nucleotide sequences present in large number of copies in the genome (repetitive DNA) give more intense hybridization signals than single-copy DNA sequences upon hybridization in which complete genomic DNA is used as a probe^38^. Colony hybridization was conducted under 65 °C in 20 mM sodium phosphate buffer pH 7.2, 20% SDS (allowing ~80% sequence identity). Washing was performed at 62 °C in 20 mM sodium phosphate buffer, 1% SDS. To detect the hybridization signal, membranes were incubated with anti-digoxigenin conjugated with alkaline phosphatase and chemiluminescent signals were induced by the addition of CDP-Star (Roche), and captured on X-ray films (Amersham). Colonies that gave most intense hybridization signals were selected and grown overnight in Luria-Bertani medium. Plasmids were isolated using Plasmid Mini Kit (QIAGEN) and their inserts were sequenced.

Sequenced inserts covered ~160,000 bp of the *Donax trunculus* genome (number of clones: 96, clone lengths between 41 bp and 9,200 bp), ~28,200 bp of the *Ruditapes decussatus* genome (number of clones: 36, clone lengths between 41 bp and 4,500 bp) and ~51,100 bp of the *R. philippinarum* genome, (number of clones: 62, clone lengths between 32 bp and 6,000 bp). Bioinformatic ‘all-*versus*-all’ blast sequence search within each set of genomic sequences yielded several groupings, some of them being identified as containing TRIM elements based on the structural features (TDRs, inner domains, PPTs).

### PCR amplification

According to the sequence of TRIM elements detected in *Donax trunculus*, a primer pair: TDR F – TTTACAGCCGCCACACAAGC and TDR R – CCTAGCACCGGCTTAAGCGG was constructed, located at the beginning and at the end of the TDR domains. PCR amplification was performed on genomic DNAs of bivalve species in order to obtain additional copies of the element. Initial denaturation was conducted at 94 °C for 5 min followed by 35 cycles at 94 °C for 30 s, 55 °C for 30 s, 72 °C for 30 s, and a final extension at 72 °C for 7 min. Prominent bands corresponding to sizes of the TRIM element or single TDR domains were gel-extracted using Qiagen Kit and PCR products were cloned into pGEM-T and pGEM-T Easy Vector Systems (Promega).

### Dot blot hybridization

The genomic abundance of TRIM elements was determined by dot-blot analysis. Serial dilutions of genomic DNAs and positive controls (TDR domains derived from TRIM elements of all examined species) were spotted onto positively charged nylon membranes (Roche). As negative controls, we used serial dilutions of unrelated DNA sequence holding just short fragments of similarity to TRIM elements, plasmid DNA without any insert and genomic DNA of the unrelated insect species *Tenebrio molitor*. The amounts of spotted DNA are given in Supplementary Table S1. Hybridization was performed with a digoxigenin-labeled probe derived from the TRIM elements of all examined species. It was conducted at 65 °C in 20 mM sodium phosphate buffer (pH 7.2), 20% SDS, 1 mM EDTA and 0.5% blocking reagent. Post-hybridization washes were performed at 62 °C in 0.1 × SSC, 1% SDS, allowing sequences with >75% of similarity to remain paired. Signals were detected with alkaline phosphatase conjugated anti-digoxigenin antibodies (Roche) using CDP-Star (Roche) as a substrate. Signals were quantified using ImageJ (https://imagej.nih.gov/ij/) program.

### Sequence analysis

All cloned fragments were sequenced at Macrogen Inc. (Korea). Representative sequences (the variants most similar to the consensus) of the biv-TRIM element for each of the 11 species were submitted to NCBI GenBank database, obtaining accession numbers MK069472 - MK069482 and clones presented in Fig. 1 hold accession numbers: MN219566 - MN219582, KU682293.1, KY400517.1, KC981681.1. Sequences AM851852, AM851181, AM870504.1, AM870526, AM871943.1 and AM873763.1 were retrieved from the EST database. Sequence alignments and editing were performed with Geneious 9.1.7 program (Biomatters Ltd.). Eukaryotic tRNA database from tRNA-Scan SE web server (http://lowelab.ucsc.edu/GtRNAdb/blast.html) was used in search for PBS sites. In order to further the search for PBS sites, already characterized and publicly available *Magallana* (*Crassostrea*) *gigas* tRNA sequences were downloaded from Ensembl webpage (ftp://ftp.ensemblgenomes.org/pub/metazoa/release-45/fasta/crassostrea_gigas). Following that, identification of tRNAs from *Ruditapes phillipinarum* and *Mytilus galloprovincialis* (species used in this study with available genomic data) was performed. tRNAs of *Magallana gigas* were blasted against the scaffolds of *R. phillipinarum* and *M. galloprovincialis* (BLAST search parameters: blastn; word_size 7; all other parameters set as default), and all the contigs showing similarity were extracted. They have been scanned with tRNAscan SE server to get a *de novo* prediction of tRNA sequences. After that, the consensus sequences of inner domains of biv-TRIMs from 11 bivalve species have been used to BLAST against the collections of tRNA sequences.

### *R. philippinarum* genomic data

The draft genome of *R. philippinarum*^24^ was checked for the presence of biv-TRIM by BLAST search using the biv-TRIM consensus sequence obtained from previous sequencing. Search was carried out with *blastn* default parameter search and e-value <1e^−10^, and only hits ≥100 bp were considered. Estimates of biv-TRIM copy number from BLAST results were obtained by the proportion of genome’s bases covered by biv-TRIM. Since the element has internal duplications (TDRs) that may lead to multiple hits on the same genomic location, genomic bases covered by biv-TRIM were calculated by merging contiguous BLAST hits into single spans using Bedtools v. 2.17^39^. Prior to clustering analysis of the elements from all the species, phylogenetic trees have been generated based on all recovered *R. philippinarum* TRIMs, not showing any significant grouping. Thus, as each selected sequence would be of equal contribution to the analysis, 10 randomly selected full-length elements from *R. philippinarum* genome (Supplementary Table S2) were used in multispecies analysis.

### Phylogenetic analyses

Phylogenetic analysis was carried out on biv-TRIM TDRs and internal domain sequences separately. Once isolated, TDRs and internal domains were aligned with Muscle algorithm, implemented in MEGA v.7.^40^, with default parameters. Phylogenetic trees were searched using the Maximum Likelihood method, with the General Time Reversible (GTR) substitution model and 100 bootstrap replicates for nodal support, included in MEGA v.7. Sequences shorter than 80 bp were not included in these analyses.

### Chromosome preparation and fluorochrome staining

Mitotic metaphase and meiotic prophase I chromosome preparations were obtained as it follows: after treating bivalve specimens with colchicine (0.005%) for 12 h, gills and gonads were excised, immersed in 50% and 25% seawater for 1 h and fixed in a mixture of 75% absolute ethanol and 25% acetic acid for 1 h. Small pieces of fixed material were then treated with 60% acetic acid to obtain cell suspensions that were dropped onto slides heated to 50 °C^41,42^.

Bivalve synaptonemal complexes were prepared from male specimens^41,43^. Meiotic cell suspensions were dropped onto glass slides, treated with 0.1 M sucrose and 0.5% Triton X-100 for 2 to 4 min, fixed with paraformaldehyde (4%) overnight, rinsed with distilled water and air-dried.

Chromosome preparations were stained with a mixture of DAPI (0.14 µg/mL) and PI (0.07 µg /mL) for 8 min, washed in tap water, air-dried and mounted with antifade (Vectashield, Vector). Slide visualization and photography were performed using a Nikon Eclipse-800 microscope equipped with an epifluorescence system. Separated images for each fluorochrome were obtained with a DS-Qi1Mc CCD camera (Nikon) controlled by the NIS-Elements software (Nikon). Merging of the images was performed with Adobe Photoshop CS2 (Adobe Systems).

### Fluorescent *in situ* hybridization

TRIM probes directly labelled by PCR with either biotin-16-dUTP (20 µM, Roche Applied Science) or digoxigenin-11-dUTP (5 µM, 10x DIG Labeling Mix, Roche Applied Science) were hybridized with mitotic metaphase chromosomes and synaptonemal complexes according to previously published methods^27,42^. Chromosome preparations, pre-treated with RNase and pepsin and post-fixed with formaldehyde, were denaturated for 2 min at 70 °C (mitotic chromosomes) or 80 °C (meiotic chromosomes) and hybridized overnight at 37 °C. For control purposes, in addition to TRIM, slides were hybridized with H3 histone gene probes. Preparations were then washed three times 5 min with 50% formamide in 2xSSC and another three times 5 min with 1xSSC. Different hybridization stringencies were tested by using washing temperatures of 37, 42, 45 and 48 °C. Biotin was detected with fluorescein isothiocyanate (FITC) conjugated avidin and biotinylated anti-avidin (Vector) whereas digoxigenin was detected with anti-digoxigenin antibodies conjugated with tetramethylrhodamine isothiocyanate (TRITC) (Sigma). Chromosome preparations were counterstained with DAPI, mounted with antifade and examined by fluorescence microscopy. Separated images for each fluorochrome were recorded and merged as indicated above.

## Supplementary information

Supplementary Files

## Data Availability

All data generated or analyzed during this study are included in this published article and its supplementary information files. Newly generated sequences have been submitted to GenBank, under the following accession numbers: MK069472 - MK069482 and MN219566 - MN219582.
