# Supplementary material for: Terminal-Repeat Retrotransposons in Miniature (TRIMs) in bivalves
Source: Sci Rep. 2019 Dec 27;9:19962. doi: 10.1038/s41598-019-56502-y (PMC6934838; doi:10.1038/s41598-019-56502-y)
Supplement: Supplementary file 1 — Supplementary Files [file 41598_2019_56502_MOESM1_ESM.docx]

**Terminal-Repeat Retrotransposons in Miniature (TRIMs) in bivalves**

Eva Šatović, Andrea Luchetti, Juan J. Pasantes, Daniel García-Souto,
Andrea Cedilak, Barbara Mantovani and Miroslav Plohl

Supplementary Information


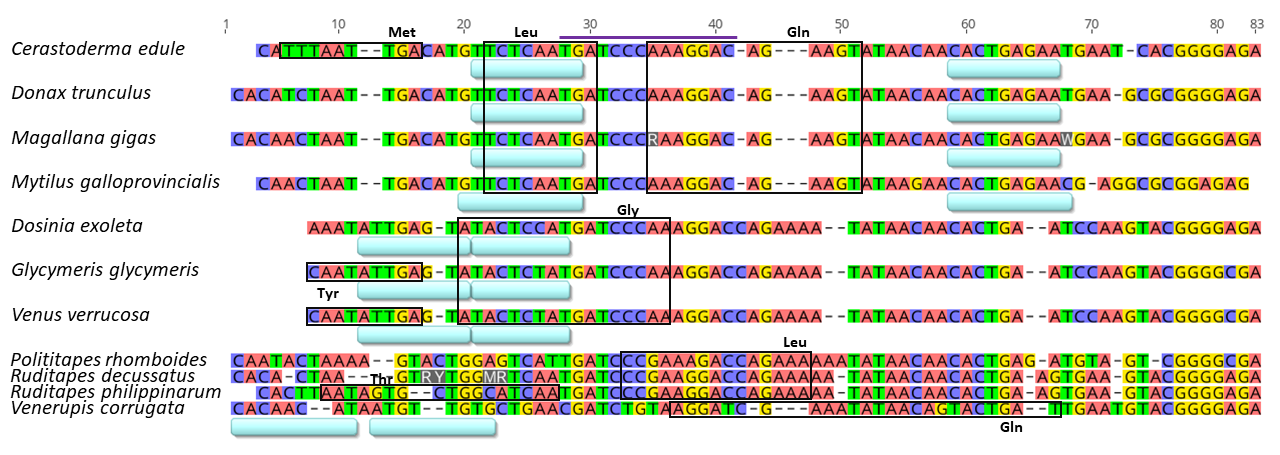


Supplementary Figure S1. Inner domains of biv-TRIM elements. Segments of inverse complementarity are labelled light blue, conserved sequence segment is marked with a purple bar above the alignment, and similarity to tRNA sequences are marked with black rectangles.


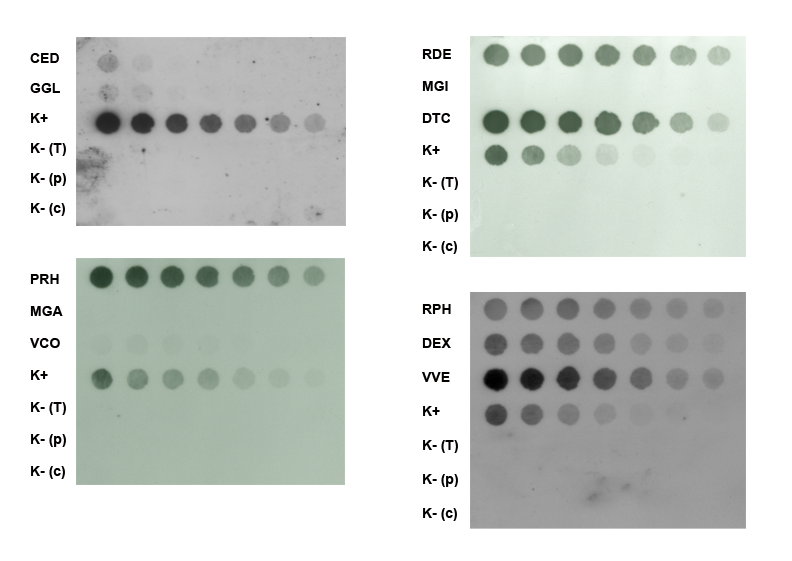


Supplementary Figure S2. Dot blot hybridization analysis on genomic DNAs from 11 bivalve species. biv-TRIM sequence was used as a positive control (K+), while negative controls were: insect *Tenebrio molitor* genomic DNA K-(T), pUC18 plasmid K-(p), *Cerastoderma edule* sequence unrelated to biv-TRIM but holding 2 short stretches of similarity to the element K-(c). CED = *C. edule*, GGL = *Glycymeris glycymeris,* RDE = *Ruditapes decussatus,* MGI = *Magallana gigas*, DTC = *Donax trunculus,* PRH = *Polititapes rhomboides*, MGA = *Mytilus galloprovincialis*, VCO = *Venerupis corrugata,* RPH = *Ruditapes philippinarum,* DEX = *Dosinia exoleta,* VVE = *Venus verrucosa*. The amounts of DNAs spotted are presented in Table S1. Full size films presented in Supplementary Figure S4.


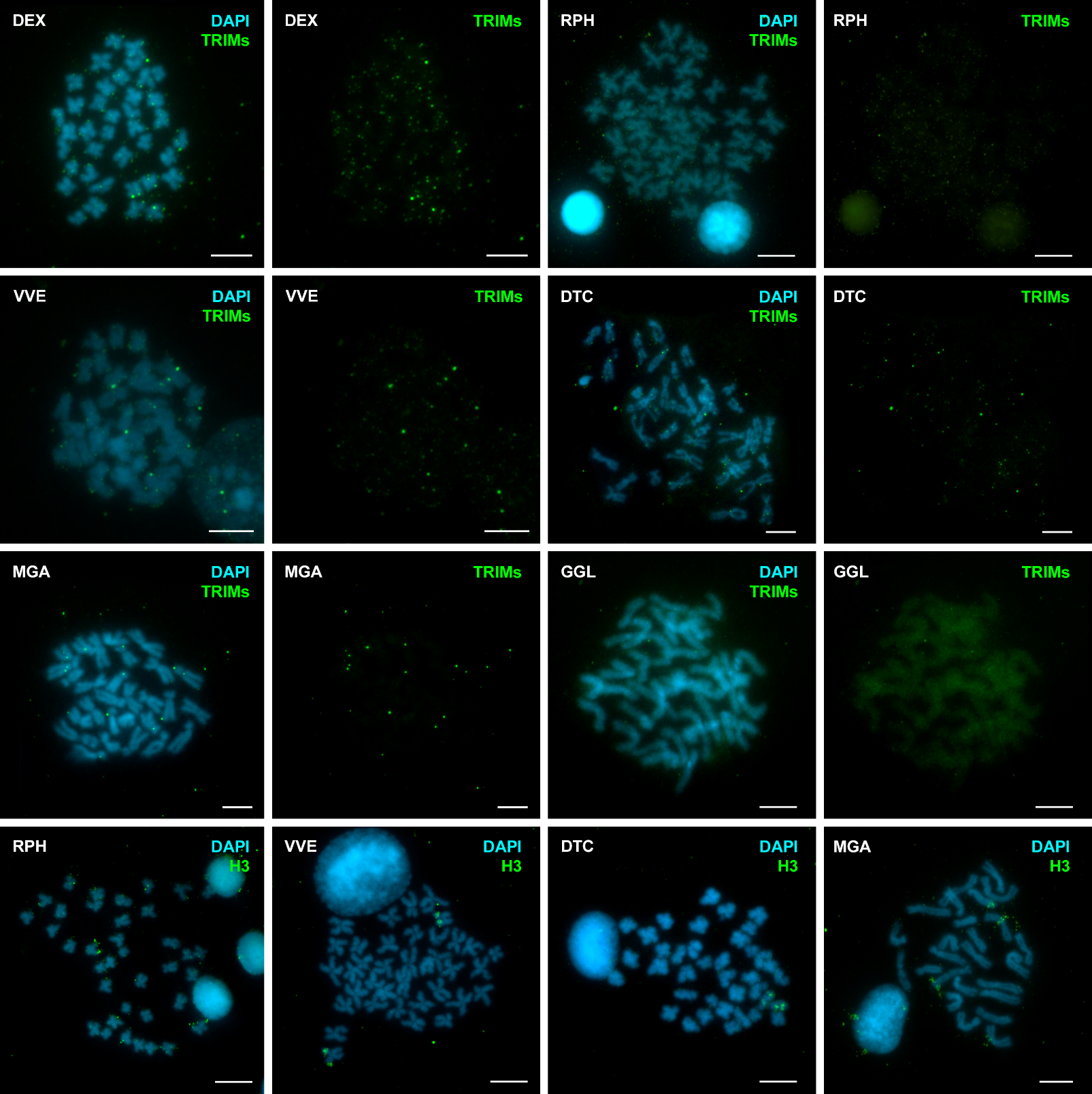


Supplementary Figure S3. Mapping of TRIM elements (green) to the chromosomes of six bivalve species counterstained with DAPI (blue). DEX = *Dosinia exoleta,* RPH = *Ruditapes philippinarum,* VVE = *Venus verrucosa,* DTC = *Donax trunculus,* MGA = *Mytilus galloprovincialis*, GGL = *Glycymeris glycymeris.* H3 histone genes were also mapped to metaphase chromosomes of several species (bottom) as a control. Scale bar = 5 µm.


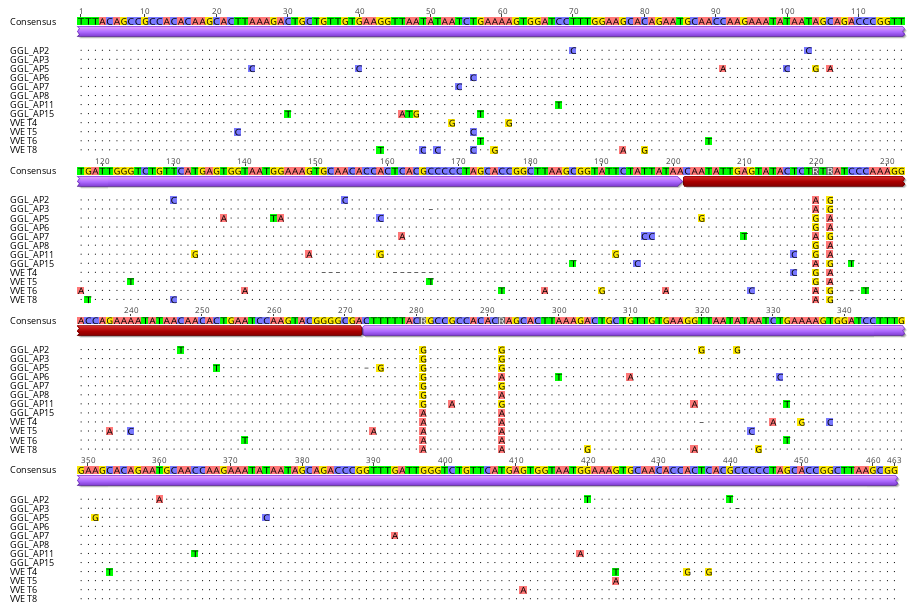


Supplementary Figure S4. Nucleotide alignment of biv-TRIM elements from *Glycymeris glycymeris* and *Venus verrucosa.* TDR domains are labeled purple and inner domain red.


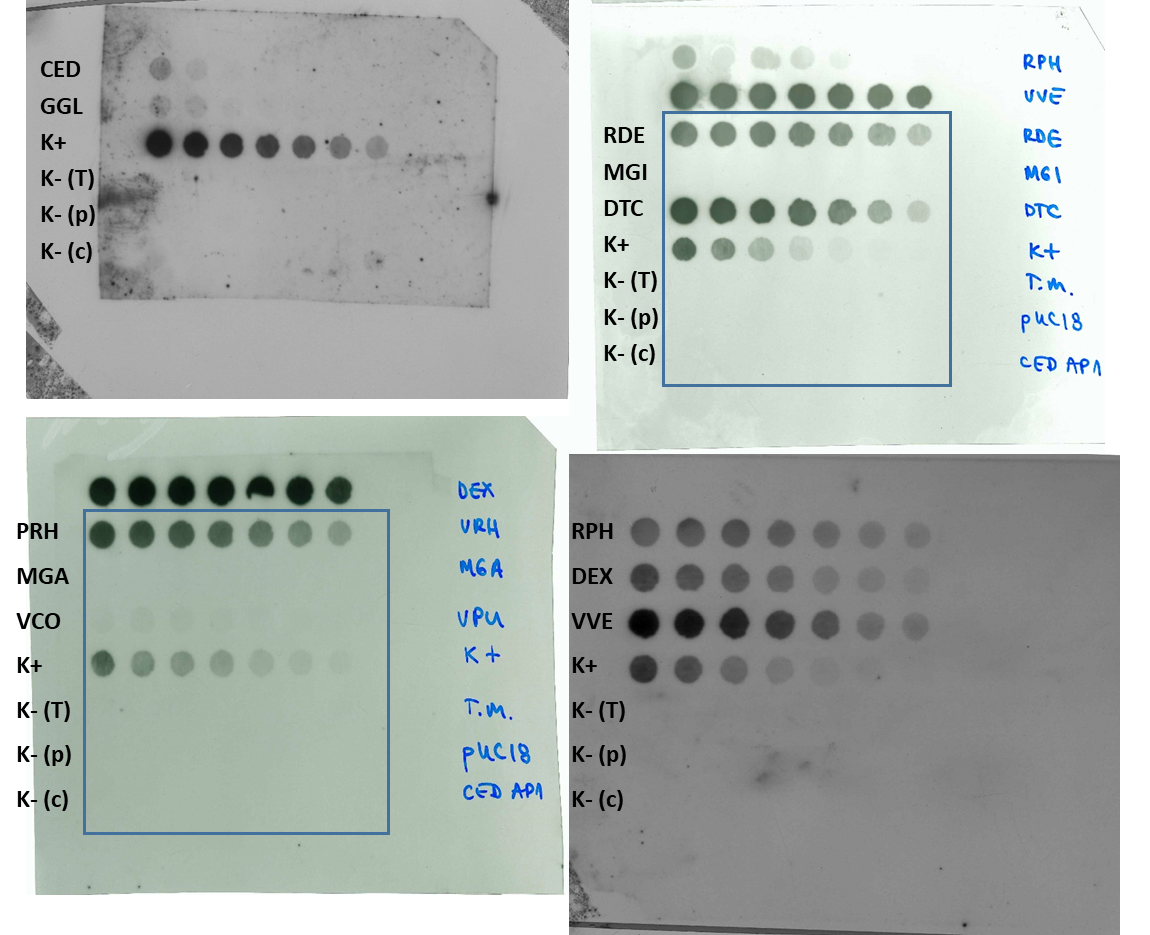


Supplementary Figure S5. Full size films from dot blot hybridization analysis presented in Supplementary Figure S2. Exposure time 30 min. Positive control (K+), negative controls K-(T), K-(p), K-(c). CED = *C. edule*, GGL = *Glycymeris glycymeris,* RDE = *Ruditapes decussatus,* MGI = *Magallana gigas*, DTC = *Donax trunculus,* PRH = *Polititapes rhomboides*, MGA = *Mytilus galloprovincialis*, VCO = *Venerupis corrugata,* RPH = *Ruditapes philippinarum,* DEX = *Dosinia exoleta,* VVE = *Venus verrucosa*.

Supplementary Table S1. Amounts of DNA spotted onto membranes for dot blot analysis

| *Cerastoderma edule*  genomic DNA (ng) | 13 000 | 6 500 | 3 250 | 1 625 | 812.5 | 406.2 | 203.1 |
| --- | --- | --- | --- | --- | --- | --- | --- |
| *Glycymeris glycymeris*  genomic DNA (ng) | 10 000 | 5 000 | 2 500 | 1 250 | 625 | 312.5 | 156.2 |
| *Polititapes rhomboides*  genomic DNA (ng) | 1750 | 875 | 437.5 | 218.75 | 109.38 | 54.69 | 27.34 |
| *Mytilus galloprovincialis*  genomic DNA (ng) | 1750 | 875 | 437.5 | 218.75 | 109.38 | 54.69 | 27.34 |
| *Venerupis corrugata*  genomic DNA (ng) | 1750 | 875 | 437.5 | 218.75 | 109.38 | 54.69 | 27.34 |
| *Ruditapes decussatus*  genomic DNA (ng) | 4000 | 2000 | 1000 | 500 | 250 | 125 | 62.5 |
| *Magallana gigas*  genomic DNA (ng) | 4000 | 2000 | 1000 | 500 | 250 | 125 | 62.5 |
| *Donax trunculus*  genomic DNA (ng) | 4000 | 2000 | 1000 | 500 | 250 | 125 | 62.5 |
| *Ruditapes philippinarum*  genomic DNA (ng) | 3500 | 1750 | 875 | 437.5 | 218.75 | 109.38 | 54.69 |
| *Dosinia exoleta*  genomic DNA (ng) | 60 | 30 | 15 | 7.5 | 3.75 | 1.875 | 0.937 |
| *Venus verrucosa*  genomic DNA (ng) | 350 | 175 | 87.5 | 43.75 | 21.875 | 10.937 | 5.468 |
| Positive control  biv-TRIM element (ng) | 0.25 | 0.125 | 0.0625 | 0.0313 | 0.0156 | 0.0078 | 0.0039 |
| Negative control  genomic DNA *T. molitor* (ng) | 0.25 | 0.125 | 0.0625 | 0.0313 | 0.0156 | 0.0078 | 0.0039 |
| Negative control  empty plasmid pUC18 (ng) | 0.25 | 0.125 | 0.0625 | 0.0313 | 0.0156 | 0.0078 | 0.0039 |
| Negative control  CED AP1 clone (ng) | 0.25 | 0.125 | 0.0625 | 0.0313 | 0.0156 | 0.0078 | 0.0039 |

Supplementary Table S2. Genomic coordinates of biv-TRIM elements isolated from the *R. philippinarum* draft genome used for phylogenetic and activity through time analysis

| **Contig** | **Position** |
| --- | --- |
| tig00000541 | 50541-51028 |
| tig00000889 | 6908-7399 |
| tig00002204 | 12205-12690 |
| tig00004913 | 89702-90194 |
| tig00017603 | 16062-16571 |
| tig00031765 | 132263-132756 |
| tig00033635 | 4770-5259 |
| tig00036144 | 62991-63482 |
| tig00044322 | 47096-47587 |
| tig00045791 | 8089-8581 |

Supplementary Table S3. Colony lift and PCR-obtained biv-TRIM sequences and their general features

| *Cerastoderma edule* | |
| --- | --- |
| CED T4 | one TDR domain, within duplication of 83 bp |
| CED AP5 | 2 TDR domains + the inner |
| CED AP17 | 2 TDR domains + the inner (small truncation on the 3' part of 3' TDR) |
| CED AP21 | 2 TDR domains + the inner |
| CED AP22 | 2 TDR domains + the inner |
| CED AP23 | 2 TDR domains + the inner |
| CED AP24 | 2 TDR domains + the inner |
| CED AP25 | 2 TDR domains + the inner |
| CED AP26 | 2 TDR domains + the inner |
| CED AP27 | 2 TDR domains + the inner |
| *Magallana gigas* | |
| MGI T1 | one TDR domain |
| MGI T2 | 2 TDR domains + the inner |
| MGI T3 | 2 TDR domains + the inner |
| *Dosinia exoleta* | |
| DEX T1 | 2 TDR domains + the inner |
| DEX T2 | one TDR domain |
| DEX T3 | 2 TDR domains + the inner |
| DEX T4 | 3 TDR domains + the inner ones (second TDR domain holding a 24 bp duplication, third TDR domain holding a 43 bp insertion) |
| DEX T5 | one TDR domain |
| DEX T6 | 2 TDR domains + the inner |
| *Glycymeris glycymeris* | |
| GGL T26 | one TDR domain |
| GGL AP1 | TDR domain (3' truncated) + second TDR domain (5' truncated), no inner domain |
| GGL AP2 | 2 TDR domains + the inner |
| GGL AP3 | 2 TDR domains + the inner |
| GGL AP5 | 2 TDR domains + the inner |
| GGL AP6 | 2 TDR domains + the inner |
| GGL AP7 | 2 TDR domains + the inner |
| GGL AP8 | 2 TDR domains + the inner |
| GGL AP11 | 2 TDR domains + the inner |
| GGL AP13 | TDR domain + inner + second TDR domain (3' truncated) |
| GGL AP15 | 2 TDR domains + the inner |
| *Mytilus galloprovincialis* | |
| MGA T1 | one TDR domain |
| MGA T2 | one TDR domain |
| MGA T3 | one TDR domain |
| MGA T5 | 2 TDR domains + the inner |
| *Ruditapes decussatus* | |
| RDE 21 | TDR domain 3' truncated + inner 5' truncated + TDR |
| RDE 22 | part of the TDR + part of the inner domain + one TDR domain |
| RDE 23 | 2 TDR domains + the inner |
| RDE 24 | one TDR domain |
| DEC 17F | part of the inner + one TDR domain (3' truncated) |
| D14 | one TDR domain (5' and 3' truncated) |
| DP33 | part of the inner + one TDR domain |
| *Ruditapes philippinarum* | |
| RPH T1 | 2 TDR domains, one at the beginning and one at the end, in different orientations |
| RPH T2 | one TDR domain holding 271 bp insertion |
| RPH T4 | TRIM at the beginning and end of the clone or 2 domain element with 385 bp insertion |
| RPH T5 | 1 TDR domain, a bit different than other RPH TDRs |
| RPH T6 | 1 TDR domain, a bit different than other RPH TDRs |
| RPH T7 | one TDR domain, within insertion of 252 bp |
| RPH T8 | one TDR domain holding 308 bp insertion |
| RPH T9 | one fragmentary TDR domain holding an 353 bp insertion |
| RPH P52 | one TDR domain, 5' truncated |
| RPH PD62 | one TDR domain, 5' truncated |
| RPH P44F | inner domain 5' truncated + TDR domain |
| RPH P10 | inner domain 5' truncated + TDR domain |
| RPH P21 | inner domain 5' truncated + TDR domain |
| RPH P39 | one TDR domain, 5' truncated |
| RPH P50F | one TDR domain, 5' truncated |
| RPH P45F | one TDR domain, 5' truncated |
| RPH P37 | one TDR domain, 5' and 3' truncated |
| *Venerupis corrugata* | |
| VCO T1 | TDR domain with a central deletion + inner + TDR |
| VCO T2 | one TDR domain |
| VCO T3 | part of the TDR + unknown + part of the inner domain + one TDR domain |
| VCO T4 | one TDR domain holding an 365 bp insertion |
| VCO T5 | part of the TDR + unknown + part of the inner domain + one TDR domain |
| VCO T6 | part of the TDR + unknown + part of the inner domain + one TDR domain |
| *Polititapes rhomboides* | |
| PRH T1 | one TDR domain, with 381 bp insertion |
| PRH T2 | 2 TDR domains + the inner |
| PRH T3 | 2 TDR domains + the inner, both TDRs holding insertions (first 100 bp, second 274 bp) |
| *Venus verrucosa* |  |
| VVE T1 | 3' truncated TDR + 5' truncated inner + complete TDR |
| VVE T2 | one TDR domain |
| VVE T3 | one TDR domain |
| VVE T4 | 2 TDR domains + the inner |
| VVE T5 | 2 TDR domains + the inner |
| VVE T6 | 2 TDR domains + the inner |
| VVE T7 | one TDR domain |
| VVE T8 | 2 TDR domains + the inner |
| VVE T9 | 2 TDR domains + the inner (with 21 bp duplication) |
| *Donax trunculus* | |
| DTC T3 | one TDR domain |
| DTC T4 | one TDR domain |
| DTC T5 | 2 TDR domains + the inner |
| DTC T6 | 2 TDR domains + the inner |
| 84-17F | one TDR domain (5' truncated)+ inner+beginning of the second TDR domain |
| DTC7Alu | TDR (5' truncated) + inner + TDR + unrelated + TDR (5' truncated and interrupted by an insertion) + inner |
| DTC13AluR | 3 TDR domains + the inner ones first and third TDR truncated) |
| DTC26Alu | TDR domain (5' truncated) + inner + second TDR domain (3' truncated) |
| DTC45Alu | one TDR domain (5' and 3' truncated) |
| DTC48Alu | TDR domain (5' truncated) + inner + second TDR domain (3' truncated) |
| DTC49Alu | TDR domain (5' truncated) + inner + second TDR domain (3' truncated) |
| DTC83Alu | TDR domain (5' truncated) + inner + second TDR domain (3' truncated) |
